# Overview of preparedness and response to COVID-19 in Ghana

**DOI:** 10.4314/gmj.v55i2s.6

**Published:** 2021-06

**Authors:** Badu Sarkodie, Franklin Asiedu-Bekoe, Dennis O Laryea, William K Ampofo, Richard O Phillips, Ali Samba, Aboagye Da Costa, Anthony Nsiah-Asare, Anarfi Asamoah-Baah, Emmanuel Odame, Sally-Anne Ohene, Yaw A Amoako, Patrick Kuma-Aboagye

**Affiliations:** 1 Ghana Health Service - Public Health Division, P. M. B. Ministries, Accra, Ghana; 2 Ghana Health Service - Disease Surveillance Department, KB 490 Korle-Bu, Accra, Ghana; 3 Department of Virology, Noguchi Memorial Institute for Medical Research, College of Health Sciences, University of Ghana. Legon, Accra, Ghana; 4 Kwame Nkrumah University of Science and Technology - Kumasi Centre for Collaborative Research, Kumasi, Ghana; 5 University of Ghana School of Medicine and Dentistry – Department of Obstetrics and Gynaecology, Accra, Ghana; 6 Presidential Taskforce on COVID-19, Office of the President, Jubilee House, Accra, Ghana; 7 Ministry of Health, P.O.Box M44 Accra, Ghana; 8 World Health Organization, Accra, Ghana; 9 Ghana Health Service Headquarters, Accra, Ghana

**Keywords:** COVID-19, Response, preparedness, pandemic, Ghana

## Abstract

The Coronavirus disease 2019 (COVID-19) outbreak in Ghana is part of an ongoing pandemic caused by the Severe Acute Respiratory Syndrome Coronavirus-2 (SARS-CoV-2). The first two cases of COVID-19 were confirmed in Ghana on 12th March 2020. COVID-19 was consequently declared a Public Health Emergency of National Concern, triggering several response actions, including enhanced surveillance, case detection, case management and contact tracing, closure of borders, suspension of international flights, ban on social gatherings and closure of schools. Preparedness and response plans were activated for implementation at the national, regional, district and community levels. Ghana's Strategic approaches were to limit and stop the importation of cases; detect and contain cases early; expand infrastructure, logistics and capacity to provide quality healthcare for the sick; minimise disruption to social and economic life and increase the domestic capacity of all sectors to deal with existing and future shocks. The health sector strategic frame focused on testing, treatment, and tracking. As of 31st December 2020, a total of 535,168 cases, including 335 deaths (CFR: 0.61%), have been confirmed with 53,928 recoveries and 905 active cases. All the regions have reported cases, with Greater Accra reporting the highest number. The response actions in Ghana have seen high-level political commitment, appropriate and timely decisions, and a careful balance of public health interventions with economic and socio-cultural dynamics. Efforts are ongoing to intensify non-pharmaceutical interventions, sustain the gains made so far and introduce COVID-19 vaccines to reduce the public health burden of the disease in Ghana

**Funding:**

None declared

## Introduction

The Coronavirus disease 2019 (COVID-19) outbreak in Ghana is part of an ongoing pandemic caused by the Severe Acute Respiratory Syndrome Coronavirus 2 (SARSCoV-2).^1^,^2^ The virus was first identified in December 2019 in China and spread quickly to many other countries, including Ghana, with the World Health Organisation (WHO) declaring it a pandemic.^3^ Ghana reported its first two cases of COVID-19 on the 12th of March 2020.^4^ Symptoms of the disease include fever, cough, fatigue, shortness of breath, and loss of smell and taste.^5^,^6^

Most infections are asymptomatic, or patients present with mild symptoms, but some progress to a severe Acute Respiratory Distress Syndrome (ARDS), which is likely to precipitate a cytokine storm.^7^ Complications of COVID-19 include pneumonia, multi-organ failure, septic shock, and death.^8^,^9^ The incubation period is five days but may range from 2–14 days.^10^,^11^ Transmission is person to person, mainly via droplets produced by coughing, sneezing, and talking.

The droplets may fall onto surfaces, and people could become infected by touching contaminated surfaces and touching their faces afterwards.

Spread is highest during the initial three days after symptoms, but the spread is possible before the onset of symptoms and from asymptomatic individuals.^12^ Standard diagnosis is by real-time reverse transcription Polymerase Chain Reaction (RT-PCR) from samples obtained via nasopharyngeal or oropharyngeal swabs or nasal lavage.^13^ Chest Computed Tomography scan (CT scan) may be helpful, but this is not recommended for routine screening.^14^,^15^ Preventive measures include using face masks in public spaces, good cough and sneezing etiquette, frequent hand washing (with water and soap) and social/physical distancing.^13^,^16^–^18^ Vaccines for COVID-19 have been developed and are in early use. Therapy with intravenous fluids, oxygen and supporting the function of affected vital organs constitute the mainstay for treatment of COVID-19.^19^–^21^ Remdesivir have been registered for use by the Food and Drugs Authority of the United States of America.^22^

Following the declaration of COVID-19 a Public Health Emergency of International Concern (PHEIC) by the World Health Organization (WHO) on the 30^th^ of January 2020, several measures were instituted in Ghana as part of preparedness for COVID-19.^23^ The subsequent declaration of COVID-19 as a pandemic by the WHO on the 11^th^ March 2020 and the confirmation of first two cases in Ghana on the 12^th^ of March 2020 led to the declaration of COVID-19 as a Public Health Emergency of National Concern by Minister for Health, triggering a series of emergency preparedness activities to prevent and protect against COVID-19 in Ghana. This report provides an overview of preparedness and response to COVID-19 pandemic in Ghana over the period January to December 2020.

### Preparedness

Preparations were initially health sector driven and included infrastructure, equipment, logistics and human resource capacity development and enhancement, public education and sensitisation, public and stakeholder engagements. There were press releases and media engagements to solicit the media's support, collaboration, and participation in providing updates and information to the public. Key activities for preparedness included the training of healthcare workers on the disease based on existing knowledge. Training also focussed on surveillance using developed case definition on COVID-19, Infection Prevention and Control for diseases spread by droplet infection and the appropriate use of Personal Protective Equipment (PPE).

At the regional and district levels, preparedness and response plans for public health emergencies were revised, and Public Health Emergency Management Committees (PHEMCs) were activated. Rapid response teams were also activated and trained. The National Technical Coordinating Committee (NTCC) and the Emergency Operations Centre (EOC) were activated at the National level. The NTCC was constituted by Ministries, Departments and Agencies with a direct role in the response. The membership of the NTCC also included international organisations such as the WHO; Research institutions such as the Noguchi Memorial Institute for Medical Research and School of Public Health at the University of Ghana. The EOC was the operational arm of the NTCC and had four thematic areas- Surveillance, Laboratory, Case Management and Risk Communication. The NTCC was responsible for coordinating the preparedness and response for COVID-19 with the EOC leading in the implementation of preparedness and response plans at the national level. At the regional and district levels, the respective PHEMCs and Rapid Response Teams (RRTs) played the roles of the NTCC and EOC, respectively. All regions and districts activated their respective RPHEMCs and District Public Health Emergency Management Committees (DPHEMC) with daily meetings.

Other preparedness actions were the development of case definitions for COVID-19 based on the WHO recommendations. Ghana adopted an extremely sensitive case definition to reduce the risk of missing a case. A health declaration form was adapted from the Ebola Virus Disease (EVD) form for use at all points of entry (PoEs). Appropriate testing kits were also provided to facilitate confirmation of COVID-19 cases in Ghana. The procurement of other logistics such as PPEs was also undertaken. Additional measures implemented as part of preparedness for COVID-19 included the use of thermal scanners and non-contact thermometers at POEs, the introduction of health declaration forms at POEs, restrictive entries (non-entry from countries with more than 200 cases), and ban on government officials from travelling.

### Response to COVID-19 in Ghana

The Government of Ghana's response was driven by three key principles: All of Society Approach, All of Government Approach, and Data and Science.

Initial response actions to the confirmation of COVID-19 in Ghana were the closure of borders to neighbouring countries (Togo, Cote d'Ivoire, and Burkina Faso) and suspension of international flights from 22^nd^ March 2020; a partial lockdown in the Greater Accra and Greater Kumasi Metropolitan areas from 30^th^ March 2020 for three weeks; the banning of social gatherings including religious activities and funerals, and the closure of schools.

### Legal and Political Framework for Response

Ghana's response actions harnessed the appropriate legal framework, including the Public Health Act 2012 (Act 851) of 2012, which grants powers to the Minister for Health in Public Health emergencies; the Imposition of Restrictions Act 2020 (Act 1012); Executive Instrument (EI) 68 of 2020 on the 17^th^ of April 2020; and EI 164 on 15^th^ June 2020. These provided the legal basis for restricting movements and social gatherings and mandatory wearing of face masks, among other measures. The response was also driven by a political framework based on the involvement of all ministries and agencies across all levels of governance from the community, through the districts, regions, to the national level. At the national level, the health sector, in collaboration with other sectors whose function impact on the response, together with development partners (multi-lateral and bilateral), activated the national public health emergency plan 24 and focused attention on the six (6) areas of the national pandemic preparedness and response plan that sought to:

1. Limit and stop the importation, detect and contain the virus
2. Slow down and manage community spread
3. Provide adequate medical and psychosocial care for COVID-19 cases
4. Strengthen Governance, Coordination and Accountability of COVID-19 Response
5. Minimise the impact of COVID-19 on Social & Economic Life
6. Increase Domestic Capacity and Self-Reliance including building and strengthening capacity for health research and Innovations

The coordination of the COVID-19 pandemic response at the national level was subsequently elevated to the Presidency with daily emergency operation meetings chaired by His Excellency the President of Ghana with the Vice President, entire Cabinet, and a team of experts in attendance. Leadership by the President provided assurance, a sense of ownership and commitment at the highest level in preparedness for the pandemic response in Ghana. An Inter-Ministerial Presidential Taskforce on COVID-19 was set up chaired by the President of Ghana. The President initiated a whole-of-government approach including Ministries of Health, Information, Interior, Communication, Education, Local Government, Trade and Industries, Environment and Sanitation, Environment Science and Technology, and Aviation. Each ministry was assigned specific tasks in the national COVID-19 response effort. The President appointed a Coordinator for COVID-19 at the Presidency and established a command centre for COVID-19.

A team of public health experts, medical laboratory scientists and expert Physicians were involved in the rigorous assessment and review of data to inform decision and policy. Processes were started for early and frequent engagements with institutions such as the Schools of Public Health, the Ghana Academy of Arts and Sciences, Professional Groupings including the Ghana Medical Association, Non-Governmental Organisations (NGOs) and Civil Society Organisations (CSOs) such as Occupy Ghana.

### Laboratory Testing as Part of the Response

At the time of confirmation of the first two cases of COVID-19 in Ghana, only two laboratories, the Noguchi Memorial Institute for Medical Research (NMIMR) and the Kumasi Centre for Collaborative Research in Tropical Medicine (KCCR) could test for COVID-19. This presented challenges to the response with increasing numbers of suspected cases requiring testing leading to a backlog of samples. An aggressive drive to increase the capacity of the existing laboratories to test was instituted. This included recruiting additional staff and the institution of 24-hour workdays for the laboratories to reduce the backlog. In addition, other laboratories within and outside the health sector, such as the Veterinary Services Department laboratory came onboard to support testing and reduce the waiting period between sample collection and testing. Private laboratories were also assessed and accredited to provide testing for COVID-19 that further increased the capacity for testing and significantly reduced the waiting time. Thus, from two testing laboratories in April 2020, the number of laboratories capable of PCR testing for COVID-19 increased to 16 [4 private all in Greater Accra and 12 Public facilities (Greater Accra-National Public Health and Reference Laboratory, Noguchi Memorial Institute for Medical Research, Veterinary Services Department Laboratory and the Council for Scientific and Industrial Research laboratory); Ashanti (Kumasi Centre for Collaborative Research and the Komfo Anokye Teaching Hospital); Volta (University for Health and Allied Sciences); Northern (Zonal Public Health Laboratory, Veterinary Service Department laboratory), Western (Veterinary Services Department Laboratory), Upper East (Navrongo Health Research Centre Laboratory). Six GeneXpert sites for SARS-CoV-2 testing were initially established with two functional at Cape Coast Teaching Hospital and Effia Nkwanta Hospital. Other GeneXpert facilities yet to be operational are Sunyani, Bolgatanga, Wa Regional and Sefwi Wiawso Municipal Hospitals. Thirty-two more sites were identified and are to be equipped to provide GeneXpert testing services for COVID-19.

The laboratory component of the response also saw a significant increase in the human resource capacity for PCR testing in Ghana. Biomedical scientists and other laboratory staff received training from the NMIMR and the KCCR to provide PCR testing services. A laboratory network was also established at the national level, with a Coordinator appointed to oversee the work of the network, including the management of logistics for sample collection, transport and testing.

Laboratory testing policy, guidelines and job-aids were developed, and staff were trained on them for immediate use. Real-time Reverse Transcription Polymerase Chain Reaction (RT-PCR) has been the mainstay of diagnosis and laboratory confirmation. Initially, testing was based on case definition, testing of close contacts, enhanced contact tracing (community screening). Prioritised testing of suspected cases, contacts, exposed health workers, students and at-risk groups was later adopted as part of the laboratory response strategy. The testing policy required initial PCR and double exit testing to assess recovery and discharge from the caregiving pathway. Following the revision of the National discharge and recovery policy, the mandatory exit testing was stopped. The pooling method was adopted initially but was suspended when the test positivity rate increased. Rapid diagnostic testing at Kotoka International Airport (KIA) using Immuno-fluorescent assay technology was introduced as part of measures to re-open KIA. Part of the laboratory response measures included evaluating the Rapid Diagnostic Test kit by the Food and Drugs Authority (FDA) in collaboration with the Noguchi Memorial Institute for Medical Research (NMIMR). At the end of December 2020, a total of 70 test kits had been evaluated. All 70 kits did not meet the set criteria for acceptability and use in Ghana for COVID-19 testing.

### Disease Surveillance and Control measures as part of Response

Surveillance was a key component of the preparedness phase for COVID-19 in Ghana. It remains a useful component of the response phase. It includes routine surveillance through case definitions, enhanced surveillance and contact tracing to ensure early case detection and management. At the onset of the outbreak globally but before the recording of the first two cases of COVID-19 in Ghana, the working case definition adopted was:

**Suspected Case:** A person presenting with fever (>38°C) or a history of fever and symptoms of respiratory tract illness, e.g., cough, difficulty in breathing **AND** in the last 14 days before symptom onset, a history of travel to China or any other affected country

**OR**

A person with fever (>38°C) or a history of fever and symptoms of respiratory tract illness, e.g., cough, difficulty in breathing **AND** in the last 14 days before symptom onset, close contact with a person who is under investigation or confirmed for 2019-nCoV.

**Confirmed Case:** A suspected case with laboratory confirmation of 2019-nCoV

Following the confirmation of cases in Ghana and the subsequent establishment of community spread in Ghana, the case definition was appropriately amended as:

**Suspected Case:** A person presenting with fever (>38°C) or a history of fever and symptoms of respiratory tract illness e.g., cough, difficulty in breathing OR other symptoms such as loss of sense of smell, loss of sense of taste

**OR**

A person with fever (>38°C) or a history of fever and symptoms of respiratory tract illness e.g., cough, difficulty in breathing or loss of sense of taste and/or smell **AND** the last 14 days before symptom onset, close contact with a person who is under investigation or confirmed for COVID-19

**Confirmed Case**: A person with laboratory confirmation of SARS-CoV-2 with or without symptoms

The overall objective of surveillance was to limit and stop the importation of cases among foreign travellers and reduce the local spread of the disease. Several activities were implemented as part of surveillance measures, including training of port health and other PoE staff (customs, national security, immigration etc.), closure of all borders (air, land, and sea) and introduction of mandatory quarantine. Mandatory quarantine for 14 days was enforced during the early phase of the pandemic in Ghana. This involved initial laboratory testing, daily monitoring for signs and symptoms of COVID-19 and exit testing at the end of the 14-day quarantine. Out of the 1,030 passengers who were quarantined upon arrival at KIA on the 21^st^ and 22^nd^ March 2020, 105 were positive for COVID-19 (79 were detected on the initial testing, and 26 were detected on exit testing). Mandatory quarantine ceased temporarily when international flights were banned but was resumed when the government approved evacuations which lasted till 1^st^ September 2020.

Additional measures implemented to aid detection and containment of cases were the:

- Development of surveillance and contact tracing guidelines, standard operation procedures (SOPs), and job-aids
- Training of health staff on the implementation of the guidelines and SOPs
- Enhanced contact tracing (implementation of which required immediate testing of all contacts without waiting for symptoms to develop)
- Use of call centres and toll-free emergency phone lines
- Regional Rapid Response Teams were trained, provided with enablers and rendered operational
- Contact tracing was decentralised and was conducted by district teams with the support of regional and national level teams
- Surveillance Outbreaks Response Management and Analysis System (SORMAS), an open-source online mobile e-Health system that processes disease control, outbreak management procedures, disease surveillance, early detection of outbreaks and also software for management for epidemiological data, was deployed and scaled up nationwide for real-time electronic data collection and transmission
- A barcode system to link surveillance and laboratory data was developed and implemented

**Figure 1:**
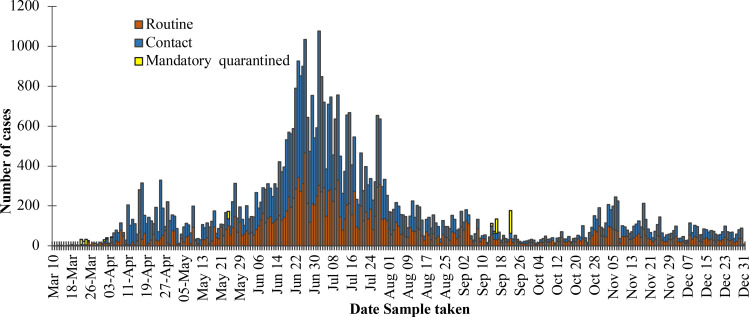
Reported cases of COVID-19 in Ghana by date and source of sample for testing, March to December 2020

Between the 12^th^ of March and 31^st^ December 2020, the surveillance system confirmed 55,168 cases of COVID-19 from a total of 672,364 tests performed, giving a positivity of 8.2%. Among these were 705 cases recorded among international arrivals at the Kotoka International Airport. A total of 335 deaths were reported among the confirmed cases of COVID giving a case fatality rate of 0.61%. Ghana recorded the first major wave of COVID-19 cases between June and August 2020 with the 2^nd^ of July 2020 recording the highest number of cases in a day (774). The trends in COVID-19 cases reported in Ghana between the 12^th^ of March and the 31^st^ of December 2020 are shown in Figure 1.

**Figure 2:**
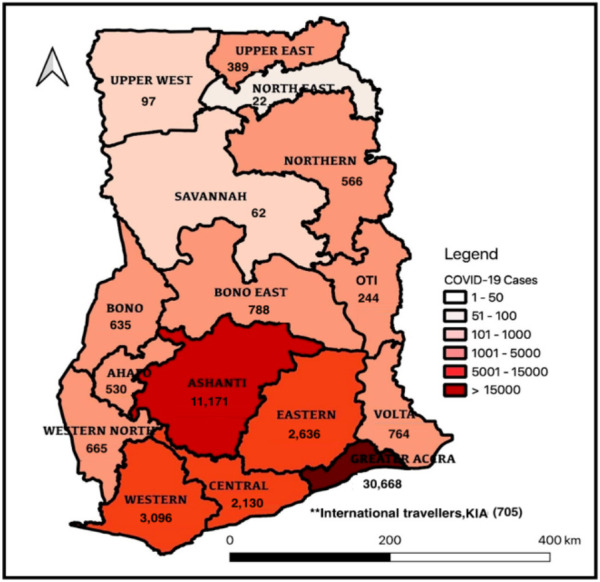
COVID-19 cases in Ghana by region, March to December 2020

All 16 regions had reported cases of COVID-19 as of the 31^st^ of December 2020. The regions reporting the most cases were the Greater Accra, Ashanti, Western, Eastern and Central Regions. The Greater Accra region alone accounted for more than 50% of the reported cases. The North East Region recorded the lowest number of cases,22. The cumulative COVID-19 cases by region from 12^th^ of March 2020 to 31^st^ December 2020 is as shown in Figure 2.

### Response measures at the Kotoka International Airport

The closure of the airport to international travel was the initial response to the pandemic in Ghana. After months of closure, the Kotoka International Airport (KIA) was re-opened on the 1^st^ of September 2020. As part of measures to ensure the safe opening of the airport and to reduce the risk of COVID-19 transmission at the airport, the following measures were undertaken:

- Re-configuration of infrastructure to ensure adequate physical and social distancing
- Logistics for hand hygiene
- Installation of operational booths for Port Health staff
- Installation and establishment of an electronic data collection system for the health declaration form (HDF) and network connection of the HDF with the testing laboratory at the KIA
- Workshops for airline operators on the guidelines on COVID-19 safety for international travels
- Simulation exercises on planned passenger flow for airport re-opening
- Recruitment of additional staff such as nurses, doctors, disease control officers and clinical psychologists to strengthen their capacity
- Establishment of a laboratory at KIA to conduct COVID-19 testing using immune-fluorescent antigen technology
- Establishment of a holding area for COVID-19 positive cases
- Provision of ambulances to transfer confirmed cases to designated treatment centres

Other activities implemented at KIA included completing the Electronic Health Declaration, an inspection of PCR test results from the country of origin for arriving passengers, temperature checks and disclosure of COVID-19 test results, isolation and transfer of COVID-19 Positive cases and contact tracing for passengers arriving at KIA.

### Contact Tracing as part of COVID-19 Response

For three weeks, the Enhanced Contact Tracing strategy was adopted to ensure early case detection and establish the extent of community spread, especially in high burden regions and partial lockdown areas in the Greater Accra and Greater Kumasi regions. Teams were trained, resourced, and deployed to test, track and link cases to care. All identified and traced contacts of confirmed cases during the lockdown period were contacted and tested for COVID-19. Approximately 150,000 tests were undertaken during the lockdown period. Multisector contact tracing teams involved national security, immigration, and health workers. Heat maps were developed, and these provided evidence of community spread.

### Risk Communication and Social Mobilisation

Risk communication and social mobilisation was a key thematic area in Ghana's preparedness and response strategy. The Ministry of Information led this thematic area in collaboration with the Health Promotion Division of the Ghana Health Service and the National Commission for Civic Education, among others. Several strategies were adopted as part of risk communication and social mobilisation. At the highest level, regularly scheduled broadcasts by the President of the Republic were employed to update the public on measures implemented by the government in response to the outbreak. These broadcasts by the President were also used to announce health policy initiatives and other critical non-health government interventions. The broadcasts by the President of the Republic were very insightful, and the origin of the now popular expression ‘Fellow Ghanaians’, which was a call to action by all, can be traced to those Presidential updates.

In addition to Presidential broadcasts, regular press briefings were part of the risk communication and social mobilisation strategies. The Ministry of Information, the Ghana Health Service, and other stakeholders provided the general public with scheduled media briefings. Subject matter experts and partners, including the Ghana Medical Association, Academia and the Ministry of Communications, were active participants in the press briefings at various points in time. One key message from such briefings was the refrain ‘the virus only moves when people move’, which helped emphasise the critical role of all Ghanaians during the critical lockdown period. Parliamentary briefings by the Minister of Health and/or Director-General of the Ghana Health Service were also implemented as part of the communication strategy.

The risk communication strategy also included developing and disseminating key messages on COVID-19 to the general population. Public education on COVID-19 across regions, districts and at the community level using traditional and social media have been key components of the risk communication strategy. Other strategies employed included the broadcast of COVID-19 related messages on screens at PoEs, media engagements (radio, TV, and print media) providing information on the disease and its prevention, media monitoring to identify challenges and misinformation on COVID-19, tour of media personnel to PoEs to provide them with a clear understanding of the operations of the Port Health Unit of the GHS and the engagement of other key stakeholders such as the National and Regional Houses of Chiefs.

### Case Management

Ghana adopted two main approaches towards case management. The first approach was isolating persons with COVID-19 at designated treatment centres (Ga East Municipal Hospital, University of Ghana Medical Centre (UGMC), all Teaching Hospitals, all Regional Hospitals, and some District Hospitals). The second approach was home isolation for asymptomatic and mild cases with no underlying medical conditions such as diabetes mellitus or hypertension.

In preparation for case management and care of the sick, the following were put in place to provide healthcare and appropriate health interventions for persons with COVID-19:

- Development of case management protocols, guidelines, and job-aids
- Staff training on the protocols and guidelines and capacity building, including infection prevention and control
- Expansion of infrastructure for holding suspected cases and isolation of confirmed cases in all health facilities
- Expansion of isolation centres and provision of equipment, PPEs, medication, and consumables
- Inclusion of clinical psychology services for patient support
- Re-assignment and deployment of selected staff of teaching hospitals with expertise in critical areas like intensive care and acute medicine to various treatment centres to facilitate the care of critically ill patients.

Case containment was a critical component of the Case Management strategy. Various activities were implemented to ensure containment, which is key to reduce the risk of disease spread.

The following were some critical components of measures aimed at case containment in Ghana's response to COVID-19:

- All health facilities were directed and supervised to establish and operate designated holding rooms for suspected cases of COVID-19.
- Metropolitan, Municipal and District Assemblies (MMDAs) partnered with the Ghana Health Service to provide quarantine facilities in their respective districts
- The national policy and guidelines on quarantine made provisions for mandatory and self-quarantine under supervision. Provisions for mandatory quarantine were enforced when conditions for self-quarantine could not be met.
- Public-Private partnerships were used to secure more facilities (including hotels, hostels, and clubhouses) for quarantine and isolation (with designated Isolation centres such as Pentecost Convention Centre (PCC), TUC (buildings in Accra and other regions), and the Ghanaman Centre of Excellence (by Ghana Football Association) in Prampram)
- Designated Treatment Centres included Tema General hospital, Ga East Municipal Hospital, Greater Accra Regional Hospital, Korle-Bu Teaching Hospital, Kumasi South, Komfo Anokye Teaching Hospital, Frimpong Boateng Medical Centre, and the Nyaho Medical Centre) Newly constructed Ghana Infectious Diseases Centre (100 bed capacity) at Ga East Municipal Hospital, Accra

The National Ambulance Service supported the response by providing ambulances at vantage locations, including the Kotoka International Airport, to transport suspected and confirmed cases. The drone delivery services were also employed in the delivery of test samples.

The health system also set out to ensure prompt linkage to care for schools. To improve school health efficiency and quality, the schools were linked with the District Health Management Teams (DHMT) and nearest health facilities. All senior high schools were supported to establish or upgrade their infirmaries, and these were linked to health facilities in the respective districts. Training of heads of schools, teachers and school health staff was undertaken.

### Logistics and Infrastructure expansion

A strategic effort was initiated for incremental procurement and stockpiling of essential supplies of PPEs. Measures were also started for local production of PPEs. More PCR machines, laboratory testing kits, infection prevention and control logistics such as hand washing facilities and equipment were procured and distributed. An active drive was initiated to expand infrastructure by identifying and converting facilities such as Ga East hospital to a treatment centre. Other facilities used as treatment centres were the Debrah Ward at 37 Military Hospital, University of Ghana Medical Centre (UGMC) and Frimpong Boateng Medical Centre in the Ashanti region. With these measures, the bed capacity for case management, which was 13 (with eight at Tema General Hospital and five at Greater Accra Regional Hospital), now increased to about 1,000 beds, including Intensive Care Units (ICUs) and High Dependency Units (HDUs).

### Partnerships and Collaboration

involvement of private health facilities in the management of public health emergencies saw significant improvement as several private health facilities and laboratories have been accredited and licensed to provide COVID-19 testing and manage cases to complement the efforts of government facilities.

The private sector support of Ghana's response extended to establishing the first Ghana Infectious Disease Centre (GIDC) at the Ga East Municipal Hospital. This well-equipped centre was constructed and furnished with funds solely from the private sector and handed over to the Government of Ghana to manage COVID-19 cases. This was a useful addition to Ghana's COVID-19 response and had the potential to be used to manage any future outbreaks of infectious diseases.

Non-governmental organisations were also involved in the public education drive for COVID-19, including the development and dissemination of information on COVID-19 safety and the provision of billboards and other communication media. The private sector contributed significantly to the Ghana COVID-19 National Trust Fund established by the government to provide a channel for resources from the non-governmental sector to be pooled in support of the government's efforts towards COVID-19 response. Resources mobilised by the Fund have been useful in the COVID response supporting several activities, including procurement of personal protective equipment and other essential logistics.

A team led by the Ministry of Health's Policy Planning Monitoring and Evaluation Directorate mobilised resources for Ghana's COVID-19 response. One major activity of this team was developing a COVID-19 Strategic Plan and Budget (2020–2024) with an estimated budget of over 600 million USD. The plan's development involved all relevant stakeholders and included the thematic leads from the laboratory, surveillance, case management, infrastructure, risk communication and social mobilisation. This plan was the basis of the development of the first Project Appraisal Document for the World Bank, for which an initial amount of 100 million dollars was raised to cover all the thematic areas.

During the outbreak, mentorship and partnership with academia and other experts were also established. Furthermore, retired clinical and public health experts were engaged to provide technical support to the COVID-19 response. The retired consultants were also involved in monitoring, mentoring and evaluation of the response. Outputs from the engagements informed policy decisions.

### Social Interventions as Part of COVID-19 Response

The government of Ghana instituted several social interventions as part of the response efforts for COVID-19. Among the many social interventions were:

*Provision of food to some residents in the Greater Accra and Greater Kumasi areas:*

During the lockdown in Greater Accra and the Greater Kumasi Areas in March and April 2020, the government provided hot meals to about 400,000 vulnerable individuals and homes.

The meals were distributed at designated points within the cities, such as the Efua Sutherland Children's Park in Accra. In addition, the government also provided dry food packs for some 470,000 families.^25^

*Absorption of Water bills and Subsidy on Electricity bills:*

The government absorbed the water bills for all consumers for six months from April 2020. In addition, public pipe-borne water stands and water tanker distribution services were provided. The government also provided a 50% rebate for all consumers of electricity and provided free electricity for customers on the lifeline tariff for nine months from April to December 2020.^26^

*Tax exemption and other social interventions for healthcare workers:* as part of response measures, the government also exempted payment of tax on the employment emoluments of healthcare workers from April 2020 to December 2020. In addition, healthcare workers working directly in the COVID-19 response classified as frontline healthcare workers received a 50% of their basic pay as a top-up to their monthly earnings from April to December 2020. Frontline healthcare workers were also provided insurance cover of up to 350,000 Ghana Cedis per worker. In addition to these, free transportation was provided for some healthcare workers in Accra, Kumasi, Tema and Kasoa.^27^

### Challenges

Despite the many useful actions undertaken as part of health sector preparedness and response to the COVID-19 pandemic in Ghana, several challenges were identified. Key among these challenges were sporadic shortages of PPEs, geographical limitations in testing for COVID-19, delays in receipt of COVID-19 tests, lack of staff to manage treatment centres and shortage of COVID-19 laboratory reagents. The shortage of PPE was addressed through the increased procurement of PPEs and the streamlining of the distribution of PPE through a sub-committee of the NTCC, the Commodities Sub-committee. This sub-committee was also instrumental in addressing laboratory reagent challenges. On staffing challenges, staff training to provide critical care services was undertaken in addition to the secondment of specialised clinical care staff from teaching hospitals to treatment centres. The expansion of laboratory testing services to include private laboratories improved the turnaround time for sample processing to about 48 hours compared to several weeks during the early phases of the pandemic, causing a reduction in the pressure brought to bear on the main testing sites, NMIMR and KCCR. The expansion of laboratory testing services also improved geographic access to COVID-19 testing, although a significant proportion of testing laboratories are in Accra. Some of the interventions to alleviate the pandemic's adverse social and financial challenges, like distribution of food during lockdown, had operational challenges resulting in dissatisfaction.

The numerous useful lessons from Ghana's response to the COVID-19 pandemic must be further studied and integrated into routine preparedness planning in Ghana. Key issues such as multisectoral collaboration, high-level political commitment, mobilising whole of government and society, private sector involvement and continuous engagement of the general population are useful, and high impact approaches for pandemic response and management of other public health emergencies.

## Conclusions

Emerging and re-emerging infections pose major threats to global health security. The COVID-19 pandemic has demonstrated the need for adequate and updated preparedness, with the availability of minimum core capacity for preparedness and response to epidemics. It is always difficult to predict when and where the next pandemic will emerge. Pandemic management and complex public health emergencies require multiple disciplines, sectors, and government apparatus. Additionally, good governance and leadership are crucial for effective, efficient, and successful management of pandemics and other complex public health emergencies, and Ghana's experience has demonstrated this.

## References

[1] Astuti I, Ysrafil (2020). Severe Acute Respiratory Syndrome Coronavirus 2 (SARS-CoV-2): An overview of viral structure and host response. Diabetes Metab Syndr.

[2] Yuen K-S, Ye Z-W, Fung S-Y, Chan C-P, Jin D-Y (2020). SARS-CoV-2 and COVID-19: The most important research questions. Cell Biosci.

[3] World Health Organisation WHO Director-General's opening remarks at the media briefing on COVID-19 - 11 March 2020.

[4] Ghana Health Service COVID-19 Updates | Ghana.

[5] United States Centers for Disease Control and Prevention Coronavirus Disease 2019 (COVID-19) - Symptoms. Centers for Disease Control and Prevention.

[6] World Health Organisation Coronavirus disease (COVID-19).

[7] Ye Q, Wang B, Mao J (2020). The pathogenesis and treatment of the ‘Cytokine Storm’ in COVID-19. J Infect.

[8] Murthy S, Gomersall CD, Fowler RA (2020). Care for Critically Ill Patients With COVID-19. JAMA.

[9] Cascella M, Rajnik M, Cuomo A, Dulebohn SC, Napoli RD (2020). Features, Evaluation, and Treatment of Coronavirus.

[10] Gandhi RT, Lynch JB, Del Rio C (2020). Mild or Moderate Covid-19. N Engl J Med.

[11] Wiersinga WJ, Rhodes A, Cheng AC, Peacock SJ, Prescott HC (2020). Pathophysiology, Transmission, Diagnosis, and Treatment of Coronavirus Disease 2019 (COVID-19): A Review. JAMA.

[12] United States Centers for Disease Control and Prevention How COVID-19 Spreads.

[13] United States Centers for Disease Control and Prevention COVID-19 guidance, tools, and resources for healthcare workers.

[14] Salehi S, Abedi A, Balakrishnan S, Gholamrezanezhad A (2020). Coronavirus Disease 2019 (COVID-19): A Systematic Review of Imaging Findings in 919 Patients. Am J Roentgenol.

[15] A P, S G, A K Comparison of influenza type A and B with COVID-19: A global systematic review and meta-analysis on clinical, laboratory and radiographic findings. Rev Med Virol.

[16] Centers for Disease Control and Prevention Recommendation Regarding the Use of Cloth Face Coverings, Especially in Areas of Significant Community-Based Transmission.

[17] World Health Organisation Advice for the public on COVID-19 - World Health Organization.

[18] United States Centers for Disease Control and Prevention Scientific Brief: SARS-CoV-2 and Potential Airborne Transmission. Centers for Disease Control and Prevention.

[19] D F, D H (2020). Q&A: The novel coronavirus outbreak causing COVID-19. BMC Med.

[20] Liu K, Fang Y-Y, Deng Y Clinical characteristics of novel coronavirus cases in tertiary hospitals in Hubei Province. Chin Med J (Engl).

[21] Wang T, Du Z, Zhu F (2020). Comorbidities and multi-organ injuries in the treatment of COVID-19. Lancet Lond Engl.

[22] United States FDA Approves First Treatment for COVID-19.

[23] World Health Organisation COVID-19 as a Public Health Emergency of International Concern (PHEIC) under the IHR | Strategic Partnership for IHR and Health Security (SPH).

[24] Ministry of Health National Strategic COVID-19 Response Plan 2020-2024.

[25] Ibrahim A Government spent ¢54.3m on cooked meals during 21-day lockdown - Ofori-Atta.

[26] Ministry of Finance Ghana Covid-19 Alleviation and Revitalization of Enterprises Support.

[27] Ministry of Health Ghana (2020). Government Provides Free Transport for Health Workers.

